# Measuring the cognitive resources consumed per second for real-time lie-production and recollection: a dual-tasking paradigm

**DOI:** 10.3389/fpsyg.2015.00596

**Published:** 2015-05-07

**Authors:** Chao Hu, Kun Huang, Xiaoqing Hu, Yanshuo Liu, Fang Yuan, Qiandong Wang, Genyue Fu

**Affiliations:** ^1^Department of Psychology, Zhejiang Normal UniversityJinhua, China; ^2^Applied Psychology and Human Development Department, University of TorontoToronto, ON, Canada; ^3^State Key Laboratory of Precision Spectroscopy, East China Normal UniversityShanghai, China; ^4^Department of Psychology, Northwestern UniversityEvanston, IL, USA; ^5^Department of Psychology, Hangzhou Normal UniversityHangzhou, China

**Keywords:** lying, lie-detection, cognitive effort, dual-tasking, eye-hand coordination

## Abstract

This research report presents a novel method of dual-tasking lie-detection. Novel software “Follow Me” was invented for a concurrent eye-hand coordination task during truth-telling/lying. Undergraduate participants were instructed to tell truths on questions about undergraduate school whereas they were instructed to tell lies on interview questions about graduate school, pretending they were graduate students. Throughout the experiment, they operated the “Follow Me” software: moving the mouse pointer to follow a randomly-moving dot on a computer screen. The distance between the mouse pointer tip and the dot center was measured by the software every 50 ms. Frequency of distance fluctuation was analyzed as the index of cognitive effort consumed per second (i.e., “degree of cognitive effort”). The results revealed that the dominant frequency of distance fluctuation was significantly lower during encoding than during retrieving responses; and lower during lying than truth-telling. Thus, dominant frequency of distance fluctuation may be an effective index of cognitive effort. Moreover, both encoding and retrieving bald-faced lies were more cognitively effortful than truth-telling. This novel definition and measurement of degree of cognitive effort may contribute to the research field of deception as well as to many other fields in social cognition.

## Introduction

Lying is a daily routine for everybody (Bond and DePaulo, 2006) and could incur significant harm for society. Thus, lie-detection is necessary and critical in multiple contexts, such as employment screening, insurance claims, and police interrogation. However, detecting lies via observing behavioral cues is usually difficult (Bond and DePaulo, 2006; Bond and Depaulo, 2008). Recent studies demonstrated that deception detection may be improved if diagnostically useful questioning is employed (Levine et al., 2014a), especially if the interrogators are experts who are familiar with the context of deception (Levine et al., 2014b). However, diagnostic interrogation is not possible in many contexts, e.g., when diagnostic interrogation is offensive. Thus, a valid and accurate measure that could help identify liars or fabricated accounts directly in these contexts is desirable. However, there is no lie-specific physiological activity that can be unambiguously linked to lies (Lykken, 1998). Thus, some researchers focus on the psychological activity inherent to lying.

Based on theories that lying is cognitively demanding and people are inferior at multi-tasking (Pashler, 1994; Logan and Gordon, 2001), researchers have proposed that lie-detection can benefit from the manipulation of cognitive load. For example, observers were more successful at identifying liars when they were telling a lie in a chronologically reverse order, which occupied more cognitive resources than telling the same lie in chronological order (Vrij et al., 2008). Lying often involves more cognitive operations and a higher cognitive workload than does truth-telling (Johnson et al., 2003, 2008; Walczyk et al., 2003; Hu et al., 2011, 2013; Debey et al., 2012), although there were some exceptions for repeated lies (Hu et al., 2012; Van Bockstaele et al., 2012).

In spite of these promising results, a study on university students' diaries revealed that lying in everyday life is usually casual (“zero consequence”), difficult to discern, and evokes little cognitive effort (DePaulo et al., 1996). In fact, some researchers believe that lying is prevalent in everyday life because lying is less cognitively effortful than truth-telling in many situations (McCornack et al., 2014).

This dispute may be due to the potential variance in definition of “cognitive effort.” Therefore, we define “cognitive effort” as the cognitive resource consumed per unit time, instead of the total cognitive resource consumed over the whole time of truth-telling/lying. It is true that lying could be less effortful than truth-telling, and thus people often lie in order to save the total cognitive resources that would be consumed over the whole time of speech (see McCornack et al., 2014). However, lying should consume more cognitive effort per unit time than truth-telling when other relevant conditions are equal—such as the amount of speech, complexity of grammar, and the difficulty in retrieving related information from memory—because compared with truth-telling, lying involves an additional cognitive process, namely the manipulation of the retrieved information. Besides, deployment of a lie usually involves awareness of the conflict between the expression and the individual's belief, which necessarily does not exist in truth-telling. Of course, deployment of a truth (e.g., admitting an extramarital relationship) could also involve other cognitive processes (e.g., estimation of the negative outcomes and related emotion reactions) that make truth-telling more effortful than lying. Nevertheless, we can hypothesize that lying should be more effortful than truth-telling if no other mental process is involved (i.e., lack of consequences or emotional reactions).

In order to test our hypotheses, we conducted a study in which a group of participants responded to the same interview questions twice. When responding to the questions the first time, the participants encoded lies/truths, and when they responded the second time, they retrieved the previously-encoded lies/truths. We hypothesized that it should be more cognitively effortful to encode than to retrieve information, as previously suggested by other researchers (see McCornack et al., 2014). Moreover, lying should be more cognitively effortful than truth-telling in both encoding and retrieving phases. A dual-task paradigm was employed to measure the degree of cognitive effort throughout the experiment.

Based on the premises that lying draws more on attention and working memory than does truth-telling, and that a concurrent task might interfere more with the former, dual-tasking was applied in lie-detection; e.g., performing a concurrent math task during the interview task (Patterson, 2009). However, the findings were weak; and this is believed to be related to the lack of theoretical rationale for the choice of the concurrent task (Walczyk et al., 2013). Therefore, we designed a concurrent task based on our definition of cognitive effort: cognitive resources consumed per unit time.

**Figure 1:**
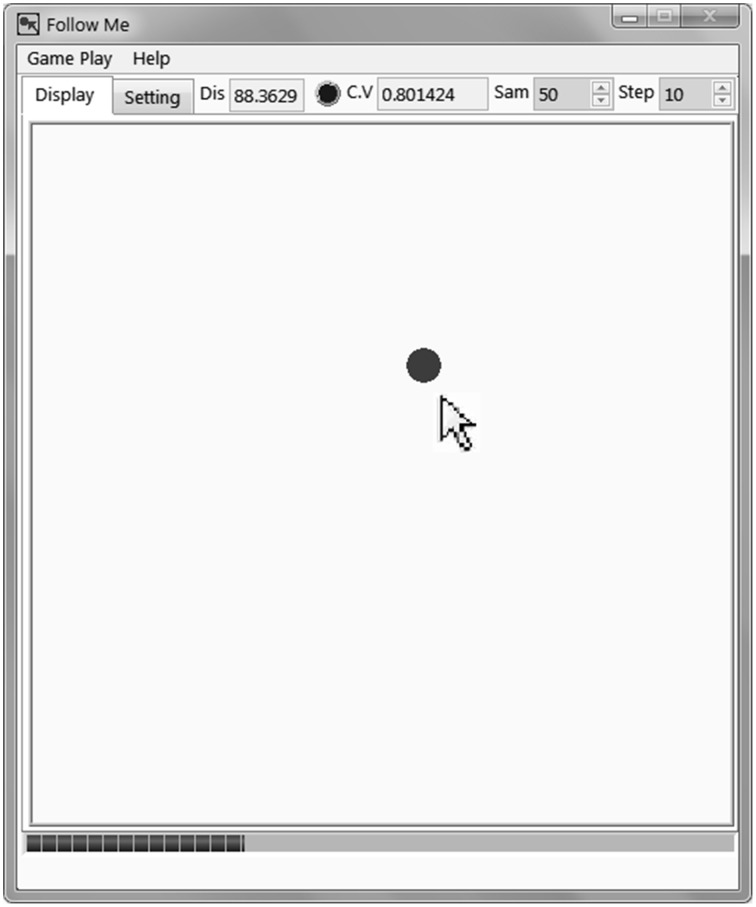
**The “Follow Me” software operation panel**.

Novel software “Follow Me” was developed to measure the cognitive resources consumed per second in an eye-hand coordination tracking task. We expected that the more cognitive resources were consumed in a concurrent task (lying/truth-telling), the fewer cognitive resources could be spent on the “Follow Me” task. Its operation panel is shown in Figure 1. The operator's task was to operate the mouse pointer to track the round red dot which is in the center of the operation panel at the beginning of the task and moves in all possible directions randomly at a stable speed after the task begins. Throughout the task, “Follow Me” records the distance between the mouse pointer tip and the dot center at a fixed frequency (1–1000 Hz).

Eye-hand coordination is a universal human capability which has been widely studied (e.g., Johansson et al., 2001), and can even be found in the newborn (von Hofsten, 1982). The “Follow Me” task is different from the majority of existing eye-hand coordination tasks in that the movement trajectory of the target was unpredictable. The obvious aim of tracking the dot is to make the distance between the mouse pointer tip and the red dot center as small as possible; thus, the average distance can serve as an index of the operator's overall performance. However, distance fluctuation frequency is the real index of cognitive effort spent upon tracking the dot per second. The distance fluctuation frequency is the number of distance fluctuation cycles per second. For example, if the distance increases from 5 to 10 pixels first, and then decreases to 0 pixels, and then increases again to 5 pixels within every second, then the frequency of the distance fluctuation would be 1 Hz at any time. In reality, this is not possible. The frequency changes over time and the variant frequencies together form a frequency spectrum. Fourier transformation was applied to analyze the dominant frequency, the most prevalent frequency throughout the duration of a truth/lie (see Welch, 1967).

Tracking the randomly-moving dot involves continuous adjustment of hand-movement direction; frequency of the adjustment determines the distance fluctuation frequency. Therefore, the distance fluctuation frequency reflects cognitive resources spent per second on the “Follow Me” task, which is covert and sensitive. This measurement is consistent with our definition of cognitive effort; that is, cognitive resources consumed per unit time.

## Methods

### Participants

One-hundred twenty-nine undergraduate students volunteered to participate in our experiment. Eight participants did not follow the experimenter's instructions correctly. Therefore, the final data were from 121 participants (56 male students, 18–25 years, Mean age = 20.5 years, *SD* = 1.22 years). They all were native Chinese and were financially rewarded 10 RMB for their participation.

### Material

Within the “Follow Me” software the radius of the red dot is set as 10 pixels. The red dot was set to move at 20 steps per second, 25 pixels per step. The “Follow Me” software measured the distance 20 times per second (All of these parameters are adjustable in the latest version of “Follow Me,” for the details, see Appendix 1).

A standard sheet of printed paper had 51 interview questions on it: 17 questions not related to school life (responses to these questions were not included in the following analyses) and 17 pairs of questions related to undergraduate/graduate school life, e.g., “What is the name of your instructor in your undergraduate/graduate school?” We matched these questions to control other factors related to the degree of cognitive effort in responding (e.g., amount and complexity of answers). Moreover, we tested whether a participant was really paying attention to our questions: if a participant's responses to a pair of questions implicated the same information, her/his data were excluded. A non-verbatim but synonymous answer was counted as the same as the original answer. No subjective judgment was required, because the answers were all concise. In this study, all of the participants were attentive and gave different responses to these matched questions (See the full list of the questions in Appendix 2).

## Procedure

The undergraduate participants were instructed to participate in a pretest for a job interview, which was intended for graduate students. The participants needed to pretend that they were graduate students and thus fabricate lies when answering questions related to “graduate school life.” The experimenter explained that the deception was limited to this hypothetical job interview and would not have any effect on real life. Personal information obtained in this study was kept private. While answering questions related to undergraduate school life, the undergraduate participants were instructed to tell the truth.

The pretest included two phases. In each phase, an assistant interviewed the participants with those 51 questions mentioned above. The orders of the questions were pseudo-randomized in both phases. After phase 1, the participants did a task unrelated to our study (i.e., Solving Raven-Matrix problems) for 5 min, and then entered phase 2 of the job interview.

The participants were randomly allocated to one of the following four groups: the Single-Single task group (*N* = 30), in which the participants did not operate the “Follow Me” task in any phase; the Dual-Single task group (*N* = 30), in which the participants operated the “Follow Me” task continually throughout phase 1; the Single-Dual task group (*N* = 30), in which the participants operated the “Follow Me” task continually throughout phase 2; the Dual-Dual task group (*N* = 31), in which the participants operated the “Follow Me” task continually throughout both phases.

The participants in the Dual-Single, the Single-Dual, and the Dual-Dual task groups practiced the “Follow Me” task before the job interview. During the practice, they responded to some questions irrelevant to the hypothetical job interview. In addition, the experimenter explained that the performance of tracking the red dot was part of the interview and they should try their best.

## Data analysis

### Consistency in responding to job interview questions

The participants responded to the same questions twice in phases 1 and 2. If their answers to a question in both phases implicated the same information, then it was counted as an instance of “consistency” in responding. The answers were all concise (e.g., names of people or places), and thus the experimenter alone coded the number of instances of consistency, as shown in Table 1.

**Table 1:** **Number of consistent responses in the interview**.

**Group Task type (sample size)**	**Truthful responses *M* (*SD*)**	**Deceptive responses *M* (*SD*)**
Single-Single (30)	12.53 (0.63)	8.10 (2.23)
Dual-Single (30)	11.27 (1.48)	6.27 (2.00)
Single-Dual (30)	11.80 (1.47)	8.10 (2.41)
Dual-Dual (31)	11.23 (1.43)	6.00 (2.48)

### Performance on the “Follow Me” task

Throughout the interview, the computer screen and the participants' verbal responses were recorded by the computer simultaneously. The recording was replayed afterward to determine the time window of the participants' responses in the data collected by the “Follow Me” software. The time when the assistant read the key word of the question (e.g., “instructor” in the question: “What is the name of your instructor in your undergraduate/graduate school years?”) was counted as the beginning of a response because the participant began to encode/retrieve truthful/deceitful information at that moment. Termination of a response was counted as its end. Data from 19 participants were not included in the following analysis because it was difficult to determine the time window of their responses in the data collected by “Follow Me.” The final analyses included 25 participants in the Dual-Single task group, 21 participants in the Single-Dual task group and 25 participants in the Dual-Dual task group. (No participant in the Single-Single group was included because no one in the Single-Single group had operated the “Follow Me” software).

**Figure 2:**
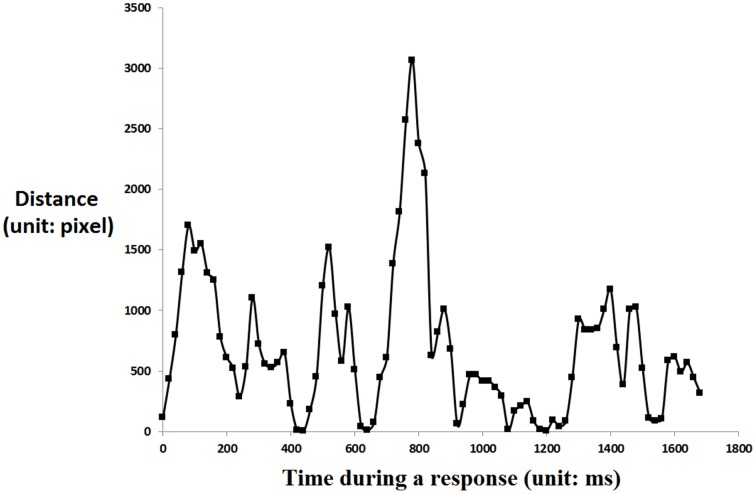
**Fluctuation of the distance from the mouse pointer tip to the dot center during a response**.

An example of the distance fluctuation during a response is shown in Figure 2. The frequency spectrum of the distance fluctuation was analyzed by Fast Fourier transformation. The dominant frequency (with the largest amplitude) in the frequency band between 0.8 and 4.4 Hz (high pass: 0.8 Hz, low pass: 4.4 Hz) was calculated for each response. (The maximum frequency in the frequency spectrum is 10 Hz.) This frequency band was selected because it captured the frequency of the operator's adjusting the direction of mouse pointer movement. In addition, maximum and mean distances were calculated during each response.

## Results

### Consistency in responding to job interview questions

In order to compare consistency in responding to different conditions, a 2 (response type: truthful/deceptive)^*^4 (task conditions: the Dual-Dual, Dual-Single, Single-Dual, Single-Single) ANOVA test was conducted on the degree of consistency. The result revealed that response type (truth/lie) effect was significant, *F*_(1, 117)_ = 489.54, *p* < 0.001, η^2^ = 0.807. Group effect was significant, *F*_(3, 117)_ = 10.06, *p* < 0.001, η^2^ = 0.205; however, the interaction between response type and group was also significant, *F*_(3,117)_ = 2.69, *p* = 0.049, η^2^ = 0.065. In order to explore the simple effect, ANOVA tests were conducted for truthful and deceptive responses separately, and both revealed significant group effects. For the truthful responses, *F*_(3, 117)_ = 6.59, *p* < 0.001, Bonferroni *Post-hoc* tests revealed that there were significant differences between the Single-Single and the Dual-Single conditions, Cohen's *d* = 1.13, *p* = 0.002; and between the Single-Single and the Dual-Dual conditions, Cohen's *d* = 1.19, *p* = 0.001. For the deceptive responses, *F*_(3, 117)_ = 7.53, *p* < 0.001, Bonferroni *Post-hoc* tests revealed that there were significant differences between the Single-Single and the Dual-Single conditions, Cohen's *d* = 0.88, *p* = 0.014; between the Single-Single and the Dual-Dual conditions, Cohen's *d* = 0.90, *p* = 0.003; between the Dual-Single and the Single-Dual conditions, Cohen's *d* = −0.84, *p* = 0.014; and between the Single-Dual and the Dual-Dual conditions, Cohen's *d* = 0.87, *p* = 0.003. Overall, participants in the Single-Single group were more consistent than those in the Dual-Single and Dual-Dual groups. Therefore, the “Follow Me” task affected consistency in responding.

Paired sample *t*-tests were conducted for each group separately. The results revealed that participants were more consistent in truth-telling than in lying; all *t* > 2.74, all *p* < 0.01. Therefore, the cognitive effort associated with lying appeared to be higher than that of truth-telling.

### Performance in the “Follow Me” task

Averaged maximum distance, averaged mean distance, and averaged dominant frequency are shown in Table 2. In order to test our hypothesis about the behavior performance in the “Follow Me” task, 2 (phase: first/second) ^*^ 2 (response type: truth/lie) repeated ANOVA analyses were performed respectively on averaged maximum distance, averaged mean distance, and averaged dominant frequency for the participants in the Dual-Dual task group (only the Dual-Dual task group had operated “Follow Me” in both phases). The effect for experimental phase was only significant for dominant frequency, *F*_(1, 23)_ = 41.02, *p* < 0.001, η^2^ = 0.64. Dominant frequency was significantly lower in phase 1 than in phase 2. Response type effect was only significant on dominant frequency, *F*_(1, 23)_ = 27.10, *p* < 0.001, η^2^ = 0.54. Dominant frequency was significantly lower during lying than during truth-telling. No interaction was significant, all *p* > 0.05.

**Table 2:** **Average distance, maximum distance, and dominant frequency in each phase (Distance unit: pixel; frequency unit: Hz)**.

**Phase\Index**	**Average distance**	**Maximum distance**	**Dominant frequency**
Encoding truths	867.58 (140.92)	4059.62 (2706.67)	1.40 (0.16)
Encoding lies	906.28 (191.69)	4155.64 (1546.39)	1.33 (0.16)
Retrieving truths	830.57 (120.85)	3231.99 (1528.90)	1.52 (0.17)
Retrieving lies	859.19 (202.62)	3636.69 (2543.24)	1.41 (0.16)

Paired sample *t*-tests on averaged maximum distance, averaged mean distance and averaged dominant frequency in phase 1 revealed that the dominant frequency during truthful responses was significantly higher than during deceptive responses, *t*_(49)_ = 5.63, *p* < 0.001, Cohen's *d* = 0.75. Other differences were not significant; all *p* > 0.05. Paired sample *t*-tests on maximum distance, mean distance, and dominant frequency in phase 2 also revealed that the dominant frequency during truthful responses was significantly higher than that during deceptive responses, *t*_(44)_ = 5.39, *p* < 0.001, Cohen's *d* = 0.79. Other differences were not significant, all *p* > 0.05.

## Discussion

Our results regarding participants' consistency in responding demonstrated that the “Follow Me” task was sufficiently cognitively demanding so as to impair the participants' performance in verbal responding. Specifically, participants performing the “Follow Me” task (the Dual-Dual task group, the Dual-Single task group) were less consistent in responding than those not performing the “Follow Me” task (the Single-Single task group).

Our hypothesis regarding the dominant frequency of distance fluctuation was confirmed. Although no significant differences in distance between the mouse pointer tip and the dot center were found, the dominant frequency of distance fluctuation during phase 1 (encoding) was significantly lower than that during phase 2 (retrieving). Moreover, the dominant frequency during lying was significantly lower than that during truth-telling. Given that participants were instructed to follow the moving dot as closely as possible, they probably paid attention to the distance between the mouse pointer tip and the dot center while failing to maintain the frequency of distance fluctuation.

Some theorists have argued that the cognitive load associated with lying will be lower when repeating a lie that previously has been articulated, compared with the production of a lie for the first time (see McCornack et al., 2014). Our results from the “Follow Me” task supported this premise. Nevertheless, even when repeating previously articulated responses, lying was still more effortful than truth-telling. This result was different from that in a previous study (see Hu et al., 2012). This may be due to the differences in defining and measuring “cognitive effort.”

Taken as a whole, these results confirm our hypothesis that the production of bald-faced lies is more cognitively effortful than that of truth, if no other mental processes are involved. Moreover, the difference between lying and truth-telling was more likely to exist in aspects of behavioral performance unnoticed by the liars, compared with those obvious aspects of behavior performance. It was easier for the participants to notice and control superficial characteristics, such as distance, rather than to detect and control latent patterns, such as frequency of distance fluctuation. This is a common pattern of behavior performance during deception. For example, when liars are controlling their facial expressions which are superficial, the lower parts of their bodies, which are less noticeable, often reveal their duplicity (for a review, see Bond and DePaulo, 2006).

Our real-time lie-detection software “Follow Me” was confirmed to be sensitive to cognitive load change. This tool is a novel invention that could inspire lie-detection practices and research fields in social cognition. It reminds us that there are always some clues that liars may fail to notice and manipulate because human cognitive resources are limited. Therefore, real-time lie-detection through observing multiple concurring behaviors may be valid in many different circumstances, including job-interviews. Above all, “Follow Me” can measure cognitive load with a high degree of temporal precision. This advantage is especially vital for many research questions for which existing technologies are not suitable. For example, lie-detection in daily life should be real-time, because partial inconsistency with facts is common in daily dialog: people usually mix lies with truths. However, measurement based on neural activity, such as fMRI, is not temporally precise enough for measuring cognitive load in real-time.

It should be noted that the deception generated in this study only involved bald-faced lies. Moreover, both the truth-telling and the lying involved zero-consequence information. The participants may not have had strong motivation for success in lying, because they wouldn't get or miss a real job because of their performance in this hypothetical job-interview. On the other hand, motivation is regarded as an important factor that affects the cognitive resources consumed by lying (Verschuere and Shalvi, 2014). Therefore, our findings may only be generalized to a narrow band of deception, such as recreational lying. Nevertheless, the “Follow Me” software could be applied to study lying in many different contexts, to explore when lying would be more involuntary and less cognitively effortful than truth-telling.

### Conflict of interest statement

The authors declare that the research was conducted in the absence of any commercial or financial relationships that could be construed as a potential conflict of interest.

## Appendix 1

### “Follow Me” software is free for the public

“Follow Me” developed together by Chao Hu, Department of Applied Psychology and Human Development, University of Toronto, and Kun Huang, State key laboratory of precision spectroscopy, East China Normal University, is free for the public, downloadable from ResearchGate of Chao S. Hu. The latest version of the software “Follow Me” has been developed by adding functions such as: 1. recording input from keyboard and mouse, so that the experimenter can mark the time windows of an event during the experiment. 2. The red dot can be set to move within a region whose boundary is invisible.

## Appendix 2

### The full list of the questions used in this study

#### A. questions related to undergraduate/graduate school life

1. Which restaurant do you usually go to in your graduate/undergraduate school?

2. What is your student number when you are in graduate/undergraduate school?

3. What is the name of the lake in your graduate/undergraduate school campus?

4. Where does you best friend in your graduate/undergraduate school come from?

5. What is the name of your instructor in your graduate/undergraduate school?

6. Have you received any scholarships in your graduate/undergraduate school?

7. What is the name of the student monitor in your graduate/undergraduate school?

8. How many credits do you need for graduation in your graduate/undergraduate school?

9. How many classmates do you have in your graduate/undergraduate school?

10. Where do you live in your graduate/undergraduate school?

11. What is the name of the dormitory monitor in your graduate/undergraduate school?

12. Where is your graduate/undergraduate school?

13. How far is your home from your graduate/undergraduate school?

14. What is the name of the student correspondent in your graduate/undergraduate school?

15. What is your favorite food in your graduate/undergraduate school?

16. How do you go back to your home from your graduate/undergraduate school?

17. What is the price of your favorite food in your graduate/undergraduate school?

#### C. questions related to personal information

1. When did you pass the level-4 English ability test?

2. What are your political beliefs?

3. Have you been a student monitor before?

4. What is your address?

5. What is your score of the Mandarin-level test?

6. What is the brand of your mobile phone?

7. Are you good at processing pictures on a computer?

8. What is your major?

9. Do you have any work experience?

10. How old are you?

11. What is your favorite sport?

12. What is your favorite song?

13. What is the name of your father?

14. What is your favorite tourist site?

15. Are you from the south of China?

16. What is your name?

17. What is your strength in your major?
